# TRPV1 channel mediates NLRP3 inflammasome-dependent neuroinflammation in microglia

**DOI:** 10.1038/s41419-021-04450-9

**Published:** 2021-12-14

**Authors:** Yahui Zhang, Baohua Hou, Peiyu Liang, Xin Lu, Yifan Wu, Xinyi Zhang, Yuanteng Fan, Yumin Liu, Taoxiang Chen, Wanhong Liu, Biwen Peng, Jun Yin, Song Han, Xiaohua He

**Affiliations:** 1 Department of Pathophysiology, School of Basic Medical Sciences, Wuhan University, Wuhan, China; 2 Medical College, Henan Polytechnic University, Jiaozuo, China; 3 Department of Neurology, Zhongnan Hospital, Wuhan University, Wuhan, China; 4 Department of Physiology, School of Basic Medical Sciences, Wuhan University, Wuhan, China; 5 Department of Immunology, School of Basic Medical Sciences, Wuhan University, Wuhan, China

**Keywords:** Neuroimmunology, Neuroimmunology

## Abstract

Multiple sclerosis (MS) is a chronic inflammatory autoimmune disease in the central nervous system (CNS). The NLRP3 inflammasome is considered an important regulator of immunity and inflammation, both of which play a critical role in MS. However, the underlying mechanism of NLRP3 inflammasome activation is not fully understood. Here we identified that the TRPV1 (transient receptor potential vanilloid type 1) channel in microglia, as a Ca^2+^ influx-regulating channel, played an important role in NLRP3 inflammasome activation. Deletion or pharmacological blockade of TRPV1 inhibited NLRP3 inflammasome activation in microglia in vitro. Further research revealed that TRPV1 channel regulated ATP-induced NLRP3 inflammasome activation through mediating Ca^2+^ influx and phosphorylation of phosphatase PP2A in microglia. In addition, TRPV1 deletion could alleviate mice experimental autoimmune encephalomyelitis (EAE) and reduce neuroinflammation by inhibiting NLRP3 inflammasome activation. These data suggested that the TRPV1 channel in microglia can regulate NLRP3 inflammasome activation and consequently mediate neuroinflammation. Meanwhile, our study indicated that TRPV1–Ca^2+^–PP2A pathway may be a novel regulator of NLRP3 inflammasome activation, pointing to TRPV1 as a potential target for CNS inflammatory diseases.

## Introduction

Multiple sclerosis is a chronic neurodegenerative autoimmune disease of the CNS that affects millions of people worldwide [1, 2]. Experimental autoimmune encephalomyelitis (EAE) is one of the most widely used animal models to study MS. It has been generally accepted that the pathogenesis of MS or EAE is due to T cell’s autoreactivity to antigens in the CNS [1–3]. However, the differentiation of pathogenic T cells is influenced by cytokines that are produced by the innate immune system, such as IL-1β, IL-6, and IL-18 [1]. Many researchers believe that the activation of microglia may act as one of the initial force in EAE pathogenesis, preceding and possibly triggering T cell differentiation and infiltration [4]. Indeed more and more studies confirm that activated microglia contribute to neurodegeneration as their number correlates with the extent of neuroinflammation and axonal damage in MS or EAE lesions [5]. Therefore, inhibiting inflammatory activation of microglia is a potential therapeutic strategy for MS.

NLRP3 inflammasome is an intracellular multiple-protein complex in the innate immune cells, which is formed by NOD-like receptor containing pyrin-domain 3 (NLRP3), apoptosis-associated speck-like protein containing CARD (ASC), and proteolytic enzyme Caspase-1 [6, 7]. NLRP3 recognizes danger signals, as well as recruiting and activating ASC and Caspase-1. Activated Caspase-1 induces the maturation and secretion of IL-1β and IL-18 [8]. Therefore, NLRP3 inflammasome regulates host inflammatory response to maintain homeostasis. However, dysregulation of this complex leads to undesirable inflammatory diseases, including inflammatory bowel disease [9], Alzheimer’s disease [10–12], amyotrophic lateral sclerosis [13], and MS [3, 14]. These studies indicated that NLRP3 inflammasome might be a potential target for the treatment of inflammatory diseases. In addition, NLRP3 can be activated by different pathogens or danger signals [15, 16]. However, how exactly these stimuli activate NLRP3 remains controversial.

Several cellular signals have been proposed to explain the activation of NLRP3 inflammasome, including disturbance of intracellular ion homeostasis (K^+^, Ca^2+^, and Cl^−^) [15, 17–22], reactive oxygen species production [23, 24], mitochondrial damage [21, 25], and lysosomal rupture [8]. The disturbance of intracellular ion homeostasis is considered to be the most likely cellular event for NLRP3 inflammasome activation. Recent studies have indicated that calcium influx or mobilization is critical for NLRP3 inflammasome activation, and TRPM2, TRPA1, and TRPV2 was the involved ion channel mediating calcium influx in the different stressed conditions [23, 24, 26–28]. However, it is unclear whether there is any other TRP channel responsible for NLRP3 inflammasome activation in microglia.

TRPV1, a prominent member of the TRP ion-channel superfamily, is a Ca^2+^-permeable channel mostly studied as a pain receptor in sensory neurons [29, 30]. It can be activated by noxious hot temperatures (>42 °C) and certain compounds such as capsaicin and acid [29]. Despite its definite role in pain sensation, more and more evidence shows that TRPV1 is also expressed in neuronal and glial cells of various brain regions, although its role is still elusive [31–34]. Some studies showed that once activated, TRPV1 mediated a variety of changes in microglia ranging from cell death to phagocytosis, cell migration, cytokine production, and ROS generation, indicating its proinflammatory role in neuroinflammation [35]. However, its role in regulating the activation of NLRP3 inflammasome is currently unclear.

In this study, we demonstrated that TRPV1 regulated NLRP3 inflammasome activation through increasing Ca^2+^ influx and the activity of phosphatase PP2A in microglia. Furthermore, we explored the role of TRPV1 in NLRP3 inflammasome-dependent EAE, and found that TRPV1 deficiency could alleviate EAE through inhibiting NLRP3 activation and rescue the neuroinflammation in mice. These findings may provide new strategies for the treatments of neuroinflammatory diseases including MS.

## Materials and methods

### Mice

All mice are on the C57BL/6 background. WT mice were bought from Beijing Vital River Laboratory Animal Technology Co., Ltd. and TRPV1-KO (TRPV1^−^^/−^) mice came from Nanjing Biomedical Research Institute. Animals were bred and kept in an SPF facility at Wuhan University Center for Animal Experiment. All animal experiments were done with approval by the Institutional Animal Care and Use Committee of Wuhan University and in accordance with institutional regulations.

### Antibodies and other reagents

Antibodies for IL-1β, caspase-1, PP2A, and phospho-PP2A (Y307) were obtained from Abcam. NLRP3 and ASC antibodies were provided by R&D Systems and Adipogen respectively. Other antibodies used in this study include: β-actin (Proteintech Group), IBA-1 (GeneTex), TRPV1 (Alomone labs), Goat Anti-Rabbit IgG-HRP (CST), Goat Anti-Mouse IgG-HRP (CST), Goat Anti-Rat IgG-HRP (Proteintech Group), Dylight 488, Goat Anti-Rabbit IgG (Abbkine), Dylight 488, Goat Anti-Mouse IgG (Abbkine), Dylight 594, Goat Anti-Rabbit IgG (Abbkine), Dylight 594, and Goat Anti-Mouse IgG (Abbkine).

PAMPs used in this study were LPS (*Escherichia coli* 0111: B4, L2630, Sigma), ATP (MedChemExpress), and Nigericin sodium salt (MedChemExpress). Chemical inhibitors and reagents include: Capsazepine (CPZ, MedChemExpress), BAPTA-AM (MedChemExpress), Okadaic acid (OA, Beyotime Biotechnology), Z-VAD-FMK (MedChemExpress), Fluo-3 AM (Dojindo), and Pluronic F-127 (Sigma).

### EAE induction

NLRP3 inflammasome-dependent EAE was induced as previously reported [31, 36]. Put simply, six- to eight-week-old female WT and TRPV1-KO C57BL/6 mice were immunized subcutaneously with an emulsion of 100 μg of MOG_35–55_ peptide (myelin oligodendrocyte glycoprotein peptide of amino acids 35–55) in 100 μl of sterile PBS and an equal volume of CFA (Complete Freund’s Adjuvant, Chondrex) containing 2 mg/ml Mycobacterium tuberculosis H37Ra. Mice also needed an intraperitoneal injection of 200 ng of pertussis toxin (Enzo Life Sciences) in 100 μl of sterile saline at the time of immunization (day 0) and 48 h later (day 2). Clinical signs of all mice were scored from onset to the peak phase of disease as follows [37]: 0, normal; 0.5, partial tail limpness; 1, complete tail limpness; 2, hindlimb and tail limpness; 2.5, paralysis of one hindlimb; 3, both hindlimbs’ paralysis; 3.5, paralysis of both hindlimbs and one forelimb; 4, hind- and forelimb paralysis or moribund; 5, death. To eliminate any diagnostic bias, mice were scored blindly.

For CPZ administration, WT EAE mice were randomly selected and subcutaneously injected with 30 mg/kg CPZ dissolved in 10% DMSO, 5% Tween-80, and 85% saline from days 0 to 21.

### Histochemical analysis

Twenty-two days post immunization, mice were anesthetized and then transcardially perfused with PBS and 4% paraformaldehyde. Lumbar spinal cord tissues were dissected, dehydrated, and embedded in paraffin blocks and further cut into 5-μm slices. Slices were stained with Luxol fast blue for assessment of demyelination and H&E for analysis of inflammatory infiltration, or incubated with antibodies against Iba-1. We analyzed EAE histopathology on cross sections and recorded digital images with a light microscope. All the data were measured by Image J software.

### Transmission electron microscopy (TEM)

About 1-mm^3^ lumbar spinal cord was immersed in 2.5% glutaraldehyde at RT for 2 h and then at 4˚C overnight, followed by gradual dehydration in ethanol and propylene oxide, and embedded in Epon. In all 50–70 nm ultrathin sections were placed onto 200 mesh copper grids and stained with 2% uranyl acetate and 0.04% lead citrate. The stained sections were observed by transmission electron microscope (Hitachi, Tokyo, magnification, x1 500). To evaluate the demyelination pathology, the myelin density and thickness were measured by Image J software.

### Cytometric bead array (CBA) for serum cytokine measurements

To analyze the serum cytokines, peripheral blood of EAE mice was collected and centrifuged at 2500 g for 10 min to obtain the serum. The levels of TNF-α and IL-1β were analyzed by CBA (mouse inflammation panel mix-and-match subpanel, Biolegend, United States) according to the manufacturer’s manual. Cytokine levels were then quantified by flow cytometry.

### Primary microglia cultures

Mixed glia cultures were prepared from WT C57BL/6 J and TRPV1-KO mouse pups as previously described [13, 23]. Briefly, 1- to 2-day-old mouse pups were killed by decapitation, and their brains were dissected. The meninges were removed, and cerebral cortexes were cut into small pieces. The tissue pieces were homogenized and then filtered by passing through a nylon-mesh cell strainer (70 μM pores, Corning, United States). DMEM/F12 (Gibco, United States) supplemented with 10% FBS was added, and the cells were resuspended, and further were seeded into T75 flasks. Cells were then incubated in a thermostatic (37˚C) humidified CO_2_ (5%) incubator. The medium was changed every 3–4 days. About 12–15 days later, the matured microglia were separated by shaking off (200 rpm) for 2 h in a constant temperature shaker (37˚C), then the cells were collected, counted and seeded in the plates.

### NLRP3 inflammasome activation

To activate the NLRP3 inflammasome, primary microglia were primed with 100 ng/ml LPS for 3 h and then stimulated with 5 mM ATP for another 30 min or 10 μM nigericin for 1 h. Both cell supernatants and lysates were collected. Supernatants were subjected to IL-1β and TNF-α analysis by an ELISA kit according to the manufacturer’s protocol. Alternatively, the supernatants were collected and concentrated by TCA precipitation followed by anti-IL-1β immunoblotting. Cell lysates were subjected to western blot assay using related primary antibodies.

### ELISA

Primary microglia were seeded into 96-well plates. After specific PAMPs or drug treatments, the cell-free supernatants were collected for measuring the IL-1β or TNF-α level using the ELISA kit (Beijing 4 A Biotech, China) by following the manufacturer’s instructions. Absorbance of sample at a wavelength of 450 nm was measured with a microplate reader (Bio Tek, United States) and the cytokine concentration was calculated by linear regression analysis.

### Western blotting

Primary microglia were seeded into 6-well plates. After specific PAMPs or drug treatments, cell lysates were collected and the protein concentration was measured by the BCA kit (Beyotime Biotechnology, China). The spinal cord tissues of EAE mice were homogenized and then centrifuged to get the supernatants (protein). About 10 μg of total proteins were separated by 12% SDS-PAGE gels and transferred onto the PVDF membranes. Membranes were incubated with primary antibodies (IL-1β, Caspase-1, p-PP2A, PP2A, and NLRP3 antibody using 1/1000 dilution, β-actin antibody using1/5000 dilution) overnight at 4 °C, and secondary antibodies (goat anti-rabbit and anti-mouse with 1/10000 dilution) for 1 h, and blots were developed by enhanced chemiluminescence (Santa Cruz Biotechnology, United States) with an imaging system (Tanon GIS system 5200, China). β-actin was used as housekeeping controls.

### Ca^2+^ imaging

Primary microglia were seeded into confocal dishes and primed with LPS for 3 h. LPS-primed microglia were then loaded with 0.5 μM Fluo-3 AM for 40 min in a humidified 5% CO_2_ incubator at 37 °C, washed 3 times in HBSS, and incubated for an additional 30 min. Then the intracellular Ca^2+^ level of the single cell was recorded and measured by confocal laser scanning microscope (Leica TCS SP2 AOBS MP, Germany) 1 min before and during ATP stimulation. The fluorescence intensity of Ca^2+^ over time was recorded and fluorescence images were collected every 3 sec. Peak Ca^2+^ fluorescence intensity was calculated as the maximal amount of Ca^2+^ accumulated in the cell after removing baseline.

### Lactate-dehydrogenase (LDH) assay

Supernatants from CPZ pretreatment and LPS-primed microglia was collected after 30 min of 5 mM ATP incubation. LDH release was quantified by LDH assay kit (Beyotime Biotechnology) according to the manufacturer’s protocol. Cytotoxicity was calculated as a percentage of LDH in total cell lysates.

### Quantitative RT-PCR

Total RNA of cultured microglia or spinal cord tissues were extracted by TRIzol™ Reagent (Invitrogen) following the manufacturer’s protocol. RNA was converted to cDNA by RevertAid First Strand cDNA Synthesis Kit (ThermoFisher Scientific). Real-time PCR was performed with CFX96 real-time (BIORAD, United States) according to the manufacturer’s protocol. The following primers were used for mouse genes: GAPDH, 5′-TCGCTCCTGGAAGATGGTGAT-3′ and 5′-CAGTGGCAAAGTGGAGATTGTTG-3′; TRPV1, 5′-CGAGGATGGGAAGAATAACTCACTG-3′ and 5′-GGATGATGAAGACAGCCTTGAAGTC-3′. The data were analyzed by the comparative cycle-threshold (CT) method, where the amount of target gene is normalized to a housekeeping gene, GAPDH.

### Immunofluorescence microscopy

Primary microglia were seeded into confocal dishes overnight. After indicated treatment, cells were fixed with 4% PFA for 15 min and then permeabilized by 0.5% Triton X-100 for 20 min. Following blocking with 10% goat serum for 1 h, cells were incubated with indicated primary antibodies overnight at 4 °C. After three washes with PBS, cells were stained with DyLight 488/594-conjugated secondary antibodies (Abbkine) for 1 h. DAPI (Roche) was used to stain nuclei. Images were recorded immediately on a Leica confocal microscopy.

For ASC speck imaging, to prevent pyroptosis and therefore loss of ASC specks, cells were pretreated with the pan-caspase inhibitor Z-VAD-FMK (50 μM, MedChemExpress) for 30 min before microscopy.

### Statistical analysis

GraphPad Prism 8.3.0 software was used to generate graphs and statistical analysis. All values are presented as mean ± S.E.M. Data with two groups were analyzed using two-tailed t test, where comparisons of multiple groups were analyzed using one-way ANOVA (one-factor analysis) followed by Newman–Keuls post-tests or two-way ANOVA (two-factor analysis) followed by Bonferroni post-tests. Clinical scores of WT and TRPV1-KO EAE mice were analyzed using Mann–Whitney U test. The incidence of WT and TRPV1-KO EAE mice was analyzed using adjusted chi-square test. *P* values < 0.05 were considered significant.

## Results

### TRPV1 is upregulated during neuroinflammation

**Fig. 1:**
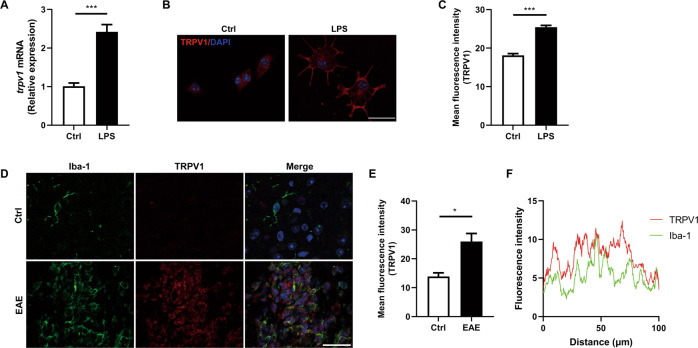
TRPV1 is upregulated during neuroinflammation. **A**–**C** Primary mouse microglia were challenged with 100 ng/ml LPS for 3 h. **A** mRNA of TRPV1 was assessed by qRT-PCR. **B** Immunostaining of TRPV1 was developed to check its expression and location change, and statistical results are shown in (**C**). **D**–**F** Immunostaining assay to check the expression of microglia TRPV1 in the spinal cord tissue of EAE mice. **D** Immunofluorescence staining of Iba-1 and TRPV1 in the spinal cord tissue of EAE mice, and statistical results are shown in (**E**). **F** Colocalization analysis of Iba-1 and TRPV1 in the spinal cord tissues of EAE mice. Representative images from *n* = 3 mice per group are displayed. Scale bar: 25μm. *p < 0.05, ***p < *0.001*.

TRPV1 is widely expressed in CNS neurons and glial cells, but whether it plays a protective or detrimental role in neuroinflammation is still controversial. To investigate this, we challenged primary mouse microglia with LPS to activate inflammation, and found that the TRPV1 mRNA level was upregulated to twice the control after LPS treatment (Fig. 1A). Meanwhile, immunostaining assay showed that the protein level of TRPV1 was also increased significantly and TRPV1 gradually translocated to the membrane from the cytoplasm after LPS challenge (Fig. 1B, C). To investigate the role of TRPV1 in neuroinflammation in vivo, we checked the expression of TRPV1 in the spinal cord tissues of EAE mice, and found that TRPV1 protein level was much higher in EAE mice than that in control mice (Fig. 1D, E) and TRPV1 was mainly located in Iba-1-positive microglia in EAE mice (Fig. 1F). Taken together, these data showed that TRPV1 expressed on the plasma membrane of microglia was upregulated during neuroinflammation in vitro and in vivo, suggesting that TRPV1 might play a functional role in regulating neuroinflammation.

### TRPV1 antagonist CPZ inhibits NLRP3 inflammasome activation in microglia

**Fig. 2:**
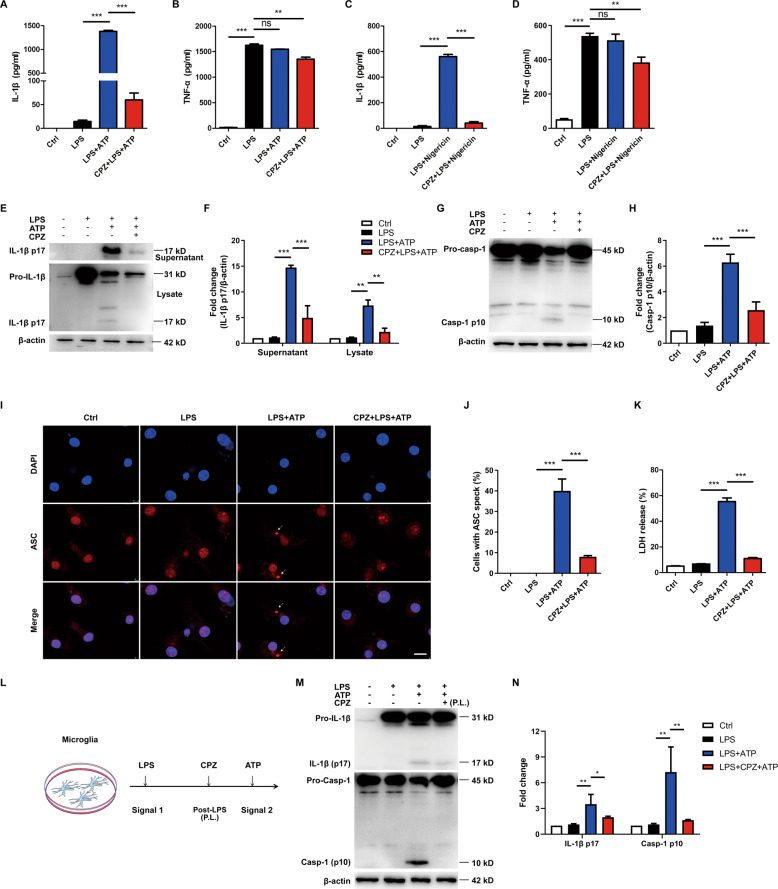
TRPV1 antagonist CPZ inhibits NLRP3 inflammasome activation in microglia. **A**–**K** Primary mouse microglia were seeded in the 96-well or 6-well plate overnight. The cells were pretreated with 10 μM Capsazepine (CPZ) for 1 h and then primed with 100 ng/ml LPS for 3 h and subsequently challenged with 5 mM ATP for 30 min or 10 μM nigericin for 1 h to activate NLRP3 inflammasome. Cleaved IL-1β p17, caspase-1 p10 and ASC specks were used as the indicators of NLRP3 inflammasome activation. **A**–**D** IL-1β and TNF-α release in the supernatants were measured using ELISA method. **E**, **F** Concentrated culture supernatants and cell lysates were immunoblotted with IL-1β p17 and Caspase-1 p10 antibody, and the results are summarized respectively in (**G**) and (**H**). Cells were fixed, permeabilized and blocked, and then stained with anti-ASC antibody. **I** ASC speck formation was detected using confocal microscopy. **J** Statistics of the percentage of cells with ASC specks. **K** Quantitative analysis of LDH released from primary microglia by LDH Cytotoxicity Assay Kit. **L**–**N** Primary mouse microglia were primed with LPS (100 ng/ml) for 3 h, and then CPZ was added for 1 h prior to the 30-min challenge of ATP to activate the NLRP3 inflammasome. **L** Drug-administration scheme. **M** Cell lysates were immunoblotted with IL-1β p17 and Caspase-1 p10 antibody, and the result is summarized respectively in (**N**). Scale bar: 10 μm. Data are representative of three independent experiments. *p < 0.05*.* **p < 0.01. ***p < 0.001.

To investigate whether TRPV1 participated in microglia NLRP3 inflammasome activation so as to regulate neuroinflammation, we first examined if Capsazepine (CPZ), a specific antagonist of TRPV1 channel, could inhibit NLRP3 inflammasome activation in vitro. Primary mouse microglia were pretreated with CPZ and then primed with LPS for 3 h, followed by an ATP challenge for 30 min. The results showed that IL-1β secretion was obviously blocked by CPZ (Fig. 2A–E) while the production of TNF-α was slightly decreased (Fig. 2B). We further examined the effect of CPZ on nigericin- (another NLRP3 inflammasome activator) induced NLRP3 inflammasome activation. Similarly, CPZ efficiently blocked IL-1β release (Fig. 2C) but still had slight effect on TNF-α secretion (Fig. 2D). Meanwhile, ATP-induced IL-1β p17 and caspase-1 p10 production in the cell lysate were suppressed by CPZ (Fig. 2E, F, G, H). ASC speck, a supramolecular oligomer of ASC, is considered to be an indicator of NLRP3 inflammasome activation. Immunostaining of the endogenous ASC in microglia showed that LPS/ATP-stimulated ASC speck formation was abolished by CPZ (Fig. 2I, J). NLRP3 inflammasome activation often led to an inflammatory cell death known as pyroptosis. Here we also examined whether CPZ could inhibit ATP-induced cell pyroptosis. Using a LDH assay reporting cell-membrane integrity, we found that CPZ completely prevented inflammasome-dependent cell death (Fig. 2K). In addition, we confirmed that CPZ had no effect on the viability of primary microglia (Fig. S1). Taken together, these results indicated that blocking TRPV1 could effectively inhibit NLRP3 inflammasome activation in microglia.

Generally, NLRP3 inflammasome activation requires two signals. A priming signal stimulates Toll-like receptor (TLR) and NF-κB pathway for the transcription of pro-IL-1β and NLRP3. An activation signal triggers assembly of inflammasome complex. To further explore whether the inhibitory effect of CPZ on NLRP3 inflammasome activation depends on TLR-NF-κB-mediated transcription, we treated LPS-primed primary microglia with CPZ prior to the 30-min challenge of ATP (Fig. 2L). After this treatment, CPZ showed little effects on pro-IL-1β expression, but was still able to inhibit ATP-induced IL-1β p17 and caspase-1 p10 production (Fig. 2M, N). This result indicated that CPZ could block NLRP3 inflammasome activation in a transcription-independent manner.

### TRPV1 mediates NLRP3 inflammasome activation in microglia

**Fig. 3:**
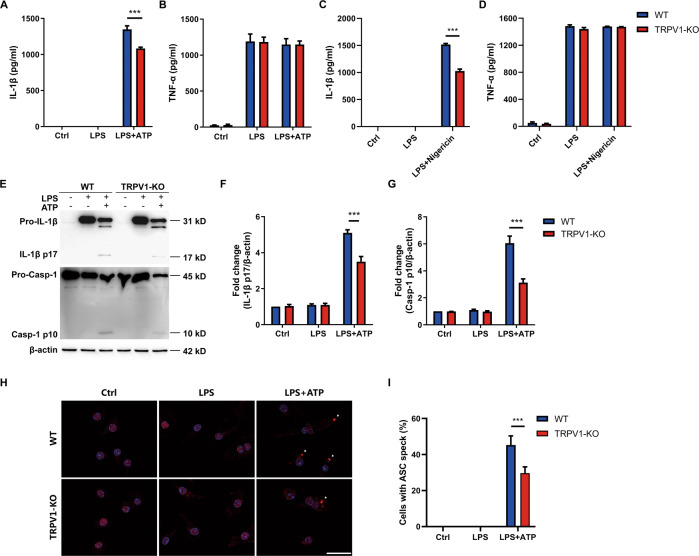
TRPV1 deletion suppresses NLRP3 inflammasome activation in microglia. Primary mouse microglia from WT and TRPV1-KO pups were seeded in 96-well or 6-well plate overnight. Then cells were primed with100 ng/ml LPS for 3 h and subsequently challenged with 5 mM ATP for 30 min or 10 μM nigericin for 1 h to activate NLRP3 inflammasome. **A–D** IL-1β and TNF-α release in the supernatants were measured using ELISA method. **E** Cell lysates were immunoblotted with IL-1β p17 and Caspase-1 p10 antibody, and the results are summarized in (**F**, **G**). Cells were fixed, permeabilized and blocked, and then stained with anti-ASC antibody. **H** ASC speck formation was detected using confocal microscopy. (**I**) Statistics of the percentage of cells with ASC specks. Scale bars: 25 μm. Data are representative of three independent experiments. ***p < 0.001.

Although we confirmed that TRPV1 antagonist CPZ could inhibit NLRP3 inflammasome activation in microglia, the function of TRPV1 in mediating NLRP3 inflammasome activation needs further evidence because of the limited specificity of CPZ on TRPV1 channel. Therefore, we examined whether TRPV1 knockout (TRPV1-KO) could suppress NLRP3 inflammasome activation. Primary microglia isolated from TRPV1-KO and WT mice were challenged with LPS plus either ATP or nigericin. As expected, TRPV1-KO microglia released less IL-1β into the supernatant than WT microglia (Fig. 3A–C), while the secretion of TNF-α was not changed (Fig. 3B–D). Immunoblot assay showed that IL-1β p17 and caspase-1 p10 production in the lysate of TRPV1-KO microglia was decreased compared to that of WT microglia (Fig. 3E, F, G). Furthermore, immunostaining showed that TRPV1-KO microglia generated less ASC specks after NLRP3 inflammasome activation by LPS plus ATP (Fig. 3H, I). Combining the results of CPZ on NLRP3 inflammasome activation, our data indicated that TRPV1 blockade or deletion could inhibit NLRP3 inflammasome activation in microglia.

### TRPV1 mediates NLRP3 inflammasome activation via Ca^2+^–PP2A signaling

**Fig. 4:**
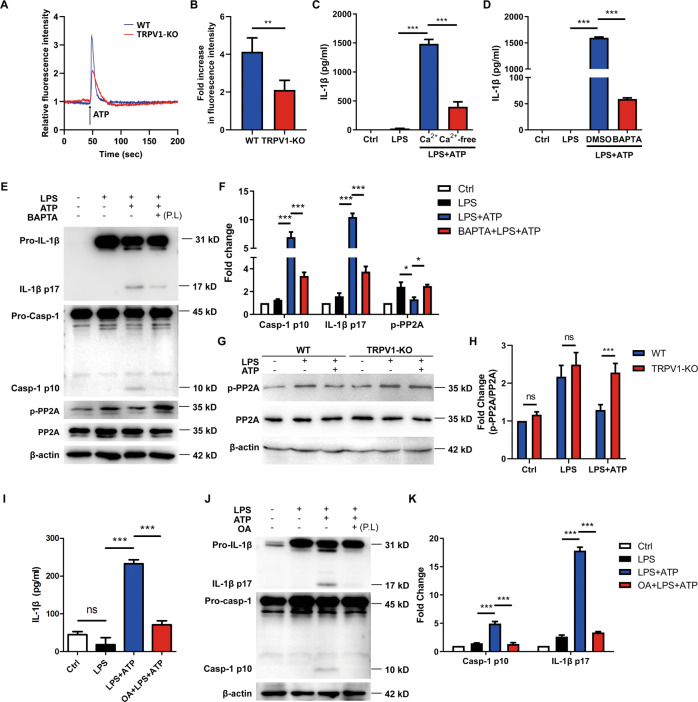
TRPV1 mediates NLRP3 inflammasome activation via Ca^2+^–PP2A signaling in microglia. **A**, **B** LPS-primed WT and TRPV1-KO microglia were loaded with 0.5 μM Fluo-3 AM for 40 min at 37 °C, followed by adding 5 mM ATP. **A** The intracellular Ca^2+^ level of the single cell was monitored 1 min before and during ATP stimulation by Leica live-cell imaging system. **B** Peak Ca^2+^ fluorescence intensity was normalized relative to the baseline intensity (data from *n* = 6 cells). **C**–**F** LPS-primed mouse microglia were treated with or without 10 μM BAPTA-AM to deplete intracellular Ca^2+^ or transferred to Ca^2+^-free (−) or Ca^2+^-containing (+) media immediately before 30-min-ATP stimulation. **C**, **D** Cell supernatants were analyzed for IL-1β by ELISA. **E** Cell lysates were immunoblotted with IL-1β p17, caspase-1 p10, and phospho-PP2A antibody, and the results are summarized in (**F**). **G**, **H** LPS-primed WT and TRPV1-KO microglia were challenged with 5 mM ATP for 30 min. **G** The cell lysates were immunoblotted with phospho-PP2A (Y307) and total PP2A antibody, and the results are summarized in (**H**). **I**–**K** LPS-primed mouse microglia were treated or not with 100 nM OA to inhibit the activity of PP2A before 30-min-ATP stimulation. **I** Cell supernatants were analyzed for IL-1β by ELISA. **J** Cell lysates were immunoblotted with IL-1β p17 and caspase-1 p10 antibody, and the results are summarized in (**K**). Data are representative of three independent experiments. *p < 0.05. **p < 0.01. ***p < 0.001.

Since we confirmed that TRPV1 can regulate NLRP3 inflammasome activation in microglia, the underlying mechanism was further investigated. Generally, TRPV1 mainly functions as Ca^2+^ channel to regulate ion homeostasis and diverse cellular processes, and blockade of Ca^2+^ influx can suppress NLRP3 inflammasome activation. Therefore, we speculated that TRPV1 might mediate microglia NLRP3 inflammasome activation by promoting Ca^2+^ influx. We measured cytosolic Ca^2+^ levels by Ca^2+^ imaging in LPS-primed WT and TRPV1-KO microglia following ATP treatment. The results showed that during ATP stimulation, cytosolic Ca^2+^ dramatically increased in a short time in WT microglia, while Ca^2+^ influx was reduced in TRPV1-KO microglia (Fig. 4A, B). We further found that incubation in Ca^2+^-free medium (Fig. 4C) and chelation of cytosolic Ca^2+^ with BAPTA-AM (Fig. 4D) both attenuated IL-1β release by ATP stimulation in LPS-primed microglia. Meanwhile, IL-1β p17 and caspase-1 p10 production in the cell lysate was also inhibited by chelation of cytosolic Ca^2+^ (Fig. 4E, F). These findings suggest that TRPV1-mediated Ca^2+^ influx is required to activate the NLRP3 inflammasome.

Next, we investigated how TRPV1-mediated Ca^2+^ influx regulates NLRP3 inflammasome activation. It was reported that the activity of phosphatase PP2A might be sensitive to cytosolic ion changes (such as K^+^ and Ca^2+^) [38, 39], and PP2A inhibition or knockdown attenuated NLRP3 inflammasome activation [40, 41]. Therefore, we speculated that TRPV1-mediated Ca^2+^ influx affected the activity of phosphatase PP2A so as to regulate NLRP3 inflammasome activation. First, we measured the activity of PP2A in the WT microglia stimulated with LPS plus ATP in the presence of BAPTA-AM to chelate cytosolic Ca^2+^. The results of immunoblot showed that the Y307 phosphorylation of PP2A (p-PP2A), which represented inactivation of PP2A, significantly increased in the presence of BAPTA-AM (Fig. 4E, F). Meanwhile, p-PP2A was also increased in the TRPV1-KO microglia stimulated with LPS plus ATP (Fig. 4G, H). These results indicated that TRPV1-mediated Ca^2+^ influx indeed regulated the activity of PP2A. Furthermore, OA (Okadaic acid), an inhibitor of PP2A, suppressed IL-1β secretion (Fig. 4I) and IL-1β p17 or caspase-1 p10 production induced by LPS plus ATP (Fig. 4J, K). Taken together, these results demonstrated that TRPV1 mediates NLRP3 inflammasome activation in the microglia via Ca^2+^–PP2A signaling.

### TRPV1 deficiency alleviates mice EAE

**Fig. 5:**
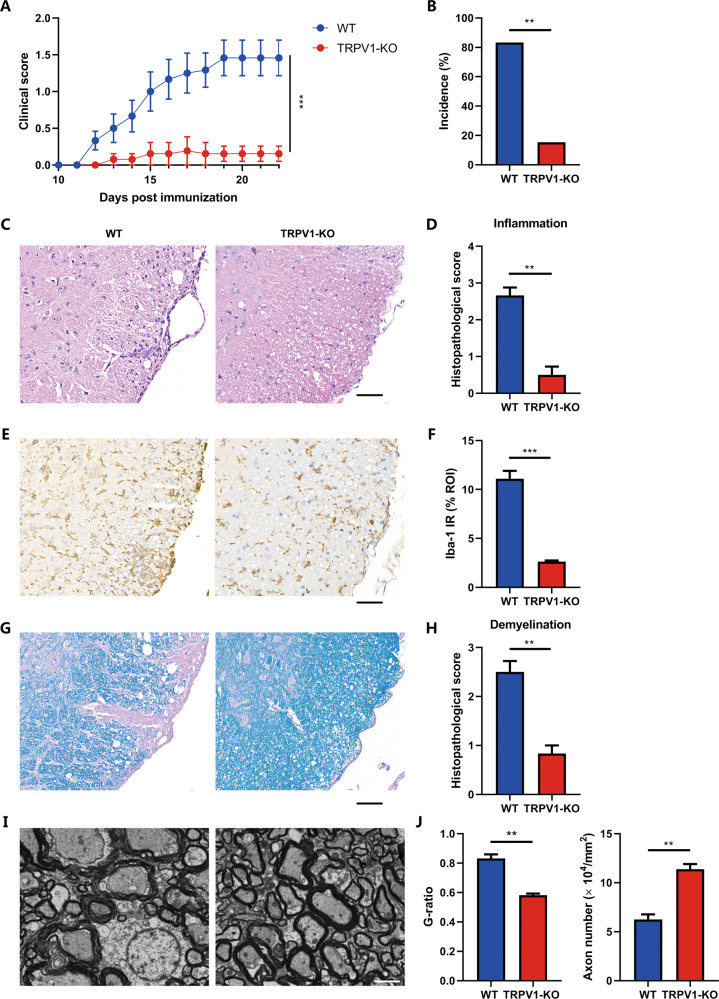
TRPV1 deficiency alleviates mice EAE. EAE was induced by active immunization of WT and TRPV1-KO C57BL/6 mice (*n* = 12 per group) with MOG_35-55_ peptide, and disease was scored from onset. **A** Clinical scores and (**B**) disease incidence for EAE from two genotypes. **C**–**H** Representative images of the spinal cord tissue sections of WT and TRPV1-KO mice after EAE induction using immunohistochemical staining. **C**, **D** Hematoxylin and eosin (H&E), (**G**, **H**) luxol fast blue (LFB), and (**E**, **F**) Iba-1 staining of lumbar spinal cord tissue sections of WT and TRPV1-KO EAE mice. Scale bar: 25 μm. Representative images from *n* = 3 mice per group are displayed. **I**, **J** Representative images of transmission electron microscopy showing the myelin sheath of spinal cord tissue from WT and TRPV1 KO mice after EAE induction. Scale bars: 5 μm. Representative images from *n* = 3 mice per group are displayed. **p < 0.01. ***p < 0.001.

Given the essential role of TRPV1 in regulating NLRP3 inflammasome activation in microglia, we further investigated whether TRPV1 could mediate NLRP3 inflammasome-dependent neuroinflammatory disease using a mice EAE model. WT and TRPV1-KO mice were immunized with MOG_35–55_ to induce EAE, and mice were euthanized at the peak phase of disease. We found that TRPV1-KO mice were rescued from EAE, developing mild and delayed disease onset and reduced clinical scores (Fig. 5A). Meanwhile, the incidence of the disease was significantly reduced in TRPV1-KO mice (Fig. 5B). Histological analysis revealed less inflammatory-cell infiltration (Fig. 5C, D) and less Iba-1-positive cell activation (Fig. 5E, F) in the lumbar spinal cord of TRPV1-KO mice. Luxol fast blue staining of myelin in the spinal cord displayed that large areas of myelin were lost in WT mice, while in TRPV1-KO mice, such lesions were small and sparse (Fig. 5G, H). TEM imaging showed that the structure of myelin sheath was loose, and myelin density or thickness was reduced in WT mice, while the disruption of myelin was obviously rescued in TRPV1-KO mice (Fig. 5I, J). Moreover, TRPV1 antagonist CPZ could also ameliorate mice EAE with reduced inflammatory infiltration and demyelination (Fig. S2A–I). In addition, we found that TRPV1-KO mice showed reduced CNS inflammation with decreased microglia activation (Fig. S3A, B, D–G) and better survival (Fig. S3C) in a LPS-induced systemic inflammation mice model. Collectively, these data confirmed that TRPV1 deficiency can alleviate neuroinflammation in vivo.

### TRPV1 deficiency inhibits NLRP3 inflammasome activation in EAE mice

**Fig. 6:**
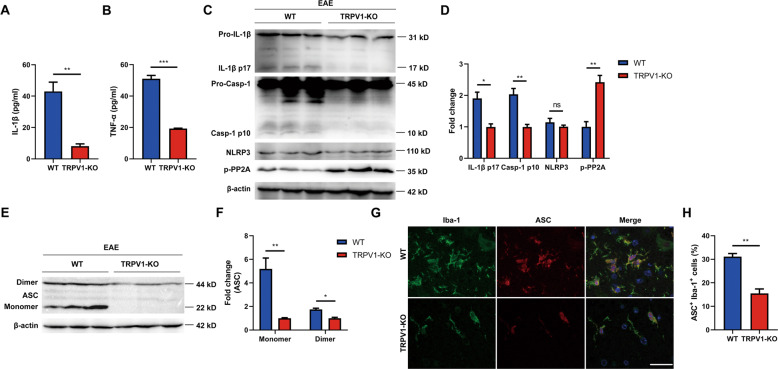
TRPV1 deficiency inhibits NLRP3 inflammasome activation in microglia of EAE mice. Serum and spinal cord tissues were collected from EAE mice at peak disease. **A**, **B** Serum cytokines, (**A**) IL-1β and (**B**) TNF-α from WT and TRPV1-KO mice, were analyzed by cytometric bead array (CBA). **C**–**F** IL-1β p17, casp-1 p10, NLRP3, p-PP2A, and ASC protein level in the spinal cord tissues from two genotypes were detected by western blot. **G**, **H** The representative image of immunofluorescence staining of Iba-1 and ASC from the lumbar spinal cord tissues of two genotypes. Representative images from *n* = 3 mice per group are displayed. Scale bars: 25 μm. *p < 0.05. **p < 0.01. ***p < 0.001.

Since we found that TRPV1 knockout can alleviate mice EAE, the effect of TRPV1 knockout on NLRP3 inflammasome activation was further investigated in EAE mice. The serum cytokines levels of EAE mice were analyzed and the results showed that IL-1β level was much lower in TRPV1-KO mice compared with WT mice (Fig. 6A), while TNF-α was slightly reduced (Fig. 6B). In addition, IL-1β p17 and caspase-1 p10 protein levels in spinal cord tissues were decreased in TRPV1-KO mice, but NLRP3 protein level was not changed between two groups (Fig. 6C, D, Fig. S4). As mentioned before, ASC oligomer was a common indicator of NLRP3 inflammasome activation. Therefore, we examined ASC oligomerization by western blotting and immunofluorescence staining in mice spinal cord tissues. The results showed that less ASC dimer (Fig. 6E, F) and ASC^+^ Iba-1^+^ cells (Fig. 6G, H) were found in TRPV1-KO mice compared with WT mice. Furthermore, we evaluated the effect of TRPV1 deficiency on PP2A activity. We found that the phosphorylation (Y307) of phosphatase PP2A was increased in TRPV1-KO mice (Fig. 6C, D). We also found that CPZ-treated EAE mice showed reduced NLRP3 inflammasome activation and PP2A activity in the spinal cord compared with EAE mice (Fig. S5A–F). These results indicated that TRPV1 knockout inhibited NLRP3 inflammasome activation in microglia of EAE mice so as to alleviate the diseases, probably through suppressing PP2A activity. These data confirmed that TRPV1 played an essential role in NLRP3 inflammasome-dependent neuroinflammatory disease.

## Discussion

In recent studies, TRPV1 has been found to be abundantly expressed in CNS, and its role in inflammatory neurodegenerative disorders has attracted more and more attention beyond its canonical role in temperature and pain sensing [32, 33, 35]. However, whether TRPV1 plays a protective or a detrimental role in neuroinflammatory disease is controversial. Some studies showed that stimulation of microglial TRPV1 controlled microglia activation and indirectly enhanced neuron toxicity [35]. TRPV1^−/^^−^ mice were protected from EAE, developing reduced clinical scores and reduced demyelination [31]. However, other studies also reported that TRPV1 receptor activation shifted the proinflammatory M1 microglia population to an anti-inflammatory M2 state and promoted neuron survival in inflammatory conditions, suggesting that TRPV1 agonist was a suitable therapeutic approach in the fight against neurodegenerative disorders [42]. Therefore, and it is still not clear how TRPV1 affects neuroinflammatory response and whether TRPV1 can regulate NLRP3 inflammasome activation in the CNS.

In the present study, we tried to illustrate the role of TRPV1 in microglia during neuroinflammation by using TRPV1-knockout mice and TRPV1 antagonist. We confirmed that the TRPV1 channel was upregulated in LPS-challenged microglia, as well as in microglia of EAE mice, and TRPV1 deficiency could inhibit NLRP3 inflammasome activation in microglia and rescue EAE mice by suppressing neuroinflammation. Our results indicated that TRPV1 played an essential role in microglial NLRP3 inflammasome activation and NLRP3 inflammasome-dependent neuroinflammatory diseases.

Generally, complete NLRP3 inflammasome activation requires two steps. A priming signal stimulates Toll-like receptor (TLR) and NF-κB pathway for the transcription of pro-IL-1β and NLRP3. An activation signal triggers assembly of inflammasome complex to activate caspase-1 and subsequently the maturation of IL-1β and IL-18 [8, 15, 19]. Our results showed that TRPV1 knockout could inhibit both ATP- and nigericin-induced NLRP3 inflammasome activation without affecting the expression of pro-IL-1β and TNF-α, suggesting that TRPV1 positively regulated NLRP3 inflammasome activation in microglia independent of NF-κB-mediated transcription. In addition, both TRPV1 knockout and CPZ could inhibit ATP-induced ASC speck formation, an indicator of inflammasome complex assembly. Therefore, we speculate that TRPV1-mediated signaling mainly participates in the activation step of NLRP3 inflammasome.

As a Ca^2+^-permeable cation channel, TRPV1can be activated by various exogenous (capsaicin, resiniferatoxin and some venom toxin) and endogenous (high temperature, acid, ATP, etc.) stimulus. Previous studies have made some conclusions regarding the role of Ca^2+^ in NLRP3 inflammasome activation [8, 18, 19, 22, 24]. So here we investigated whether TRPV1 regulated NLRP3 inflammasome activation by mediating Ca^2+^ influx. By using Ca^2+^ imaging, we found that in LPS-primed TRPV1-KO microglia, the intracellular calcium current induced by ATP increased slightly compared with WT microglia. We also noticed that TRPV1 knockout could not completely prevent ATP-induced Ca^2+^ influx to the cytoplasm and the activation of NLRP3 inflammasome (Fig. 4A, Fig. 3A–C), while deletion of cytosolic Ca^2+^ could completely prevent the activation of NLRP3 inflammasome (Fig. 4D). These results indicated that TRPV1-mediated Ca^2+^ influx was not the only source of intracellular Ca^2+^, and there might be other Ca^2+^ channels involved in Ca^2+^ influx during microglia activation.

To date, the downstream targets of Ca^2+^ signal regulating NLRP3 inflammasome activation remain to be defined, although a Ca^2+^–CaMKII–TAK1–JNK pathway in macrophages has been reported [8]. Recently, PP2A was shown to license NLRP3 inflammasome assembly via directly dephosphorylating serine 3 (serine 5 of human) of NLRP3 PYD domain [40]. PP2A is ubiquitously expressed in eukaryotic cells as a heterotrimeric protein phosphatase composed of a catalytic C subunit, a scaffolding A subunit and a regulatory B subunit. The B subunits are considered to influence enzyme activity, substrate specificity, and subcellular localization. There are five different isoforms of B subunit, including PR65, PR72, and PR130 [39, 43]. It was reported that PP2A with PR72 subunit was expressed in the brain, which had two Ca^2+^-binding sites and could be directly activated by Ca^2+^ binding [39]. Based on these studies, we proposed and further proved that TRPV1 deficiency inhibited NLRP3 inflammasome activation via reducing Ca^2+^ influx and the following inactivation of PP2A in microglia under LPS plus ATP challenge.

Consistent with our in vitro data, we found that TRPV1 played an essential role in NLRP3 inflammasome-dependent inflammatory diseases in vivo. First, by using a mice EAE model, we found that TRPV1-KO and TRPV1 antagonist-treated EAE mice displayed reduced NLRP3 inflammasome activation and less demyelination compared with WT or vehicle-treated EAE mice. In addition, TRPV1-KO mice showed better survival and reduced CNS inflammation in LPS-induced systemic inflammation mice model (Fig. S5). Surprisingly, TRPV1-KO mice displayed reduced serum TNF-α level compared with WT mice in inflammatory conditions, while TRPV1-KO had little effect on TNF-α secretion in vitro (Fig. 3B–D, E). We speculated that TRPV1-mediated intracellular signal might affect TNF-α secretion in other inflammatory cells (e.g., T cells), since it was reported that TRPV1 regulated the activation and proinflammatory properties of CD4^+^ T cells [30, 44].

In conclusion, our results revealed that TRPV1 mediated NLRP3 inflammasome activation via Ca^2+^–PP2A pathway in microglia and TRPV1 ablation could efficiently inhibit neuroinflammation. The therapy targeting TRPV1 channel may be a promising strategy for suppressing NLRP3-driven neuroinflammation.

## Supplementary information

supplementary figure legend

Figure S1

Figure S2

Figure S3

Figure S4

Figure S5

checklist

## Data Availability

The data that support the findings of this study are available from the corresponding author upon reasonable request.

## References

[1] Gris D, Ye Z, Iocca HA, Wen H, Craven RR, Gris P (2010). NLRP3 plays a critical role in the development of experimental autoimmune encephalomyelitis by mediating Th1 and Th17 responses. J Immunol.

[2] Martin BN, Wang C, Zhang C-j, Kang Z, Gulen MF, Zepp JA (2016). T cell–intrinsic ASC critically promotes TH17-mediated experimental autoimmune encephalomyelitis. Nat Immunol.

[3] Inoue M, Williams K, Gunn M, Shinohara M (2012). NLRP3 inflammasome induces chemotactic immune cell migration to the CNS in experimental autoimmune encephalomyelitis. Proc Natl Acad Sci USA.

[4] Voet S, Mc Guire C, Hagemeyer N, Martens A, Schroeder A, Wieghofer P (2018). A20 critically controls microglia activation and inhibits inflammasome-dependent neuroinflammation. Nat Commun.

[5] Zabala A, Vazquez-Villoldo N, Rissiek B, Gejo J, Martin A, Palomino A (2018). P2X4 receptor controls microglia activation and favors remyelination in autoimmune encephalitis. EMBO Mol Med.

[6] Zhou K, Shi L, Wang Y, Chen S, Zhang J (2016). Recent advances of the NLRP3 inflammasome in central nervous system disorders. J Immunol Res.

[7] Shen H-H, Yang Y-X, Meng X, Luo X-Y, Li X-M, Shuai Z-W (2018). NLRP3: a promising therapeutic target for autoimmune diseases. Autoimmun Rev.

[8] Okada M, Matsuzawa A, Yoshimura A, Ichijo H (2014). The lysosome rupture-activated TAK1-JNK pathway regulates NLRP3 inflammasome activation. J Biol Chem.

[9] Mao L, Kitani A, Hiejima E, Montgomery-Recht K, Zhou W, Fuss I (2020). Bruton tyrosine kinase deficiency augments NLRP3 inflammasome activation and causes IL-1beta-mediated colitis. J Clin Invest.

[10] Stancu IC, Cremers N, Vanrusselt H, Couturier J, Vanoosthuyse A, Kessels S (2019). Aggregated Tau activates NLRP3-ASC inflammasome exacerbating exogenously seeded and non-exogenously seeded Tau pathology in vivo. Acta Neuropathol.

[11] Heneka MT, Kummer MP, Stutz A, Delekate A, Schwartz S, Vieira-Saecker A (2013). NLRP3 is activated in Alzheimer’s disease and contributes to pathology in APP/PS1 mice. Nature.

[12] Ising C, Venegas C, Zhang S, Scheiblich H, Schmidt SV, Vieira-Saecker A (2019). NLRP3 inflammasome activation drives tau pathology. Nature.

[13] Deora V, Lee JD, Albornoz EA, McAlary L, Jagaraj CJ, Robertson AAB (2020). The microglial NLRP3 inflammasome is activated by amyotrophic lateral sclerosis proteins. Glia.

[14] Freeman L, Guo H, David CN, Brickey WJ, Jha S, Ting JP (2017). NLR members NLRC4 and NLRP3 mediate sterile inflammasome activation in microglia and astrocytes. J Exp Med.

[15] Munoz-Planillo R, Kuffa P, Martinez-Colon G, Smith BL, Rajendiran TM, Nunez G (2013). K+ efflux is the common trigger of NLRP3 inflammasome activation by bacterial toxins and particulate matter. Immunity.

[16] Song N, Liu ZS, Xue W, Bai ZF, Wang QY, Dai J (2017). NLRP3 phosphorylation is an essential priming event for inflammasome activation. Mol Cell.

[17] Green JP, Yu S, Martin-Sanchez F, Pelegrin P, Lopez-Castejon G, Lawrence CB (2018). Chloride regulates dynamic NLRP3-dependent ASC oligomerization and inflammasome priming. Proc Natl Acad Sci USA.

[18] Lee GS, Subramanian N, Kim AI, Aksentijevich I, Goldbach-Mansky R, Sacks DB (2012). The calcium-sensing receptor regulates the NLRP3 inflammasome through Ca2+ and cAMP. Nature.

[19] Murakami T, Ockinger J, Yu J, Byles V, McColl A, Hofer A (2012). Critical role for calcium mobilization in activation of the NLRP3 inflammasome. Proc Natl Acad Sci USA.

[20] Petrilli V, Papin S, Dostert C, Mayor A, Martinon F, Tschopp J (2007). Activation of the NALP3 inflammasome is triggered by low intracellular potassium concentration. Cell Death Differ.

[21] Tang T, Lang X, Xu C, Wang X, Gong T, Yang Y (2017). CLICs-dependent chloride efflux is an essential and proximal upstream event for NLRP3 inflammasome activation. Nat Commun.

[22] Vaeth M, Zee I, Concepcion AR, Maus M, Shaw P, Portal-Celhay C (2015). Ca2+ signaling but not store-operated Ca2+ entry is required for the function of macrophages and dendritic cells. J Immunol.

[23] Aminzadeh M, Roghani M, Sarfallah A, Riazi GH (2018). TRPM2 dependence of ROS-induced NLRP3 activation in Alzheimer’s disease. Int Immunopharmacol.

[24] Zhong Z, Zhai Y, Liang S, Mori Y, Han R, Sutterwala FS (2013). TRPM2 links oxidative stress to NLRP3 inflammasome activation. Nat Commun.

[25] Park S, Juliana C, Hong S, Datta P, Hwang I, Fernandes-Alnemri T (2013). The mitochondrial antiviral protein MAVS associates with NLRP3 and regulates its inflammasome activity. J Immunol.

[26] Compan V, Baroja-Mazo A, Lopez-Castejon G, Gomez AI, Martinez CM, Angosto D (2012). Cell volume regulation modulates NLRP3 inflammasome activation. Immunity.

[27] Tseng HH, Vong CT, Kwan YW, Lee SM, Hoi MP (2016). TRPM2 regulates TXNIP-mediated NLRP3 inflammasome activation via interaction with p47 phox under high glucose in human monocytic cells. Sci Rep.

[28] Wang M, Zhang Y, Xu M, Zhang H, Chen Y, Chung KF (2019). Roles of TRPA1 and TRPV1 in cigarette smoke -induced airway epithelial cell injury model. Free Radic Biol Med.

[29] Grundy L, Daly DM, Chapple C, Grundy D, Chess-Williams R (2018). TRPV1 enhances the afferent response to P2X receptor activation in the mouse urinary bladder. Sci Rep.

[30] Bertin S, Aoki-Nonaka Y, de Jong PR, Nohara LL, Xu H, Stanwood SR (2014). The ion channel TRPV1 regulates the activation and proinflammatory properties of CD4+ T cells. Nat Immunol.

[31] Paltser G, Liu XJ, Yantha J, Winer S, Tsui H, Wu P (2013). TRPV1 gates tissue access and sustains pathogenicity in autoimmune encephalitis. Mol Med.

[32] Tsuji F, Murai M, Oki K, Seki I, Ueda K, Inoue H (2010). Transient receptor potential vanilloid 1 agonists as candidates for anti-inflammatory and immunomodulatory agents. Eur J Pharm.

[33] Balleza-Tapia H, Crux S, Andrade-Talavera Y, Dolz-Gaiton P, Papadia D, Chen G (2018). TrpV1 receptor activation rescues neuronal function and network gamma oscillations from Abeta-induced impairment in mouse hippocampus in vitro. Elife.

[34] Musumeci G, Grasselli G, Rossi S, De Chiara V, Musella A, Motta C (2011). Transient receptor potential vanilloid 1 channels modulate the synaptic effects of TNF-alpha and of IL-1beta in experimental autoimmune encephalomyelitis. Neurobiol Dis.

[35] Marrone MC, Morabito A, Giustizieri M, Chiurchiu V, Leuti A, Mattioli M (2017). TRPV1 channels are critical brain inflammation detectors and neuropathic pain biomarkers in mice. Nat Commun.

[36] Inoue M, Chen PH, Siecinski S, Li QJ, Liu C, Steinman L (2016). An interferon-beta-resistant and NLRP3 inflammasome-independent subtype of EAE with neuronal damage. Nat Neurosci.

[37] Inoue M, Williams K, Oliver T, Vandenabeele P, Rajan J, Miao E (2012). Interferon-β therapy against EAE is effective only when development of the disease depends on the NLRP3 inflammasome. Sci Signal.

[38] Eil R, Vodnala SK, Clever D, Klebanoff CA, Sukumar M, Pan JH (2016). Ionic immune suppression within the tumour microenvironment limits T cell effector function. Nature.

[39] Ahn J, Sung J, McAvoy T, Nishi A, Janssens V, Goris J (2007). The B”/PR72 subunit mediates Ca2+-dependent dephosphorylation of DARPP-32 by protein phosphatase 2A. Proc Natl Acad Sci USA.

[40] Stutz A, Kolbe CC, Stahl R, Horvath GL, Franklin BS, van Ray O (2017). NLRP3 inflammasome assembly is regulated by phosphorylation of the pyrin domain. J Exp Med.

[41] Martin BN, Wang C, Willette-Brown J, Herjan T, Gulen MF, Zhou H (2014). IKKalpha negatively regulates ASC-dependent inflammasome activation. Nat Commun.

[42] Bok E, Chung YC, Kim KS, Baik HH, Shin WH, Jin BK (2018). Modulation of M1/M2 polarization by capsaicin contributes to the survival of dopaminergic neurons in the lipopolysaccharide-lesioned substantia nigra in vivo. Exp Mol Med.

[43] Clark AR, Ohlmeyer M (2019). Protein phosphatase 2A as a therapeutic target in inflammation and neurodegeneration. Pharm Ther.

[44] Bertin S, Aoki-Nonaka Y, Lee J, de Jong PR, Kim P, Han T (2017). The TRPA1 ion channel is expressed in CD4+ T cells and restrains T-cell-mediated colitis through inhibition of TRPV1. Gut.

