# Supplementary material for: TRPV1 channel mediates NLRP3 inflammasome-dependent neuroinflammation in microglia
Source: Cell Death Dis. 2021 Dec 14;12(12):1159. doi: 10.1038/s41419-021-04450-9 (PMC8671551; doi:10.1038/s41419-021-04450-9)
Supplement: Supplementary file 1 — supplementary figure legend [file 41419_2021_4450_MOESM1_ESM.pdf]

Fig. S1 CCK8 assay for analyzing the effect of CPZ on cell viability of primary microglia

Primary mouse microglia were cultured in the presence or absence of 10 μM CPZ for indicated time durations. CCK8 assay was performed to measure the effect of CPZ on cell viability of primary microglia.

Fig.S2 TRPV1 antagonist CPZ ameliorates mice EAE

C57BL/6 mice (n =12) were immunized with MOG_{35-55} to induce EAE. CPZ (30mg/kg) was subcutaneously administrated once a day from day 0 to day 21. Mean clinical scores (A), disease incidence (B) and percentage of mice scoring grade 2^+ from CPZ-treated and vehicle-treated group (C). Representative images of spinal cord sections from CPZ-treated and vehicle-treated mice after EAE induction using hematoxylin and eosin (H&E) (D), luxol fast blue (LFB) (F) and Iba-1 staining (H), and statistical results were shown respectively in (E, G, I). Scale bars: 25 μm. Representative images from at least three mice per group are displayed. *$p < 0.05$. **$p < 0.01$. ***$p < 0.001$.*

Fig.S3 Systemic inflammation is disrupted in TRPV1-KO mice

ELISA of IL-1β (A) and TNF-α (B) in the serum of WT and TRPV1-KO mice that were intraperitoneally (i.p.) injected with 10 mg/kg LPS for 4 h. n=3. (C) Survival of WT and TRPV1-KO mice subjected to 5 mg/kg LPS (i.p.) was monitored. n=6. Representative images of Iba-1 immunostaining of brain (D) and spinal cord (F) tissues from WT and TRPV1-KO mice subjected to 5 mg/kg LPS (i.p.) for 48 h, and statistical results were summarized respectively in (E, G). Scale bars: 50 μm. Representative images from at least three mice per group are displayed. *$p < 0.05$. ***$p < 0.001$.

Fig. S4 IL-1β expression in the spinal cord tissues of ctrl and EAE mice from two genotypes

WT and TRPV1-KO (KO) mice were immunized with $MOG_{35\text{-}55}$ to induce EAE. Mice were sacrificed in the peak phase of disease and the spinal cord tissues were homogenized to extract protein for western blot assay of IL-1β.

Fig.S5 CPZ blocks NLRP3 inflammasome activation in EAE mice

(A-B) Serum cytokines, IL-1β (A) and TNF-α (B) from CPZ-treated and vehicle-treated EAE mice were analyzed by cytometric bead array (CBA). (C) The representative image of immunofluorescence staining of Iba-1 and ASC from spinal cord tissue of each group, and statistical results were summarized in (D). (E) Western blot assay for IL-1β p17, casp-1 p10, NLRP3 and p-PP2A from the spinal cord tissues of each group and statistical results were summarized in (F). Representative images from at least three mice per group are displayed. Scale bars: 25 μm. *$p < 0.05$. **$p < 0.01$. ***$p < 0.001$.
