# Supplementary figures and images for: TRPV1 channel mediates NLRP3 inflammasome-dependent neuroinflammation in microglia
Source: Cell Death Dis. 2021 Dec 14;12(12):1159. doi: 10.1038/s41419-021-04450-9 (PMC8671551; doi:10.1038/s41419-021-04450-9)

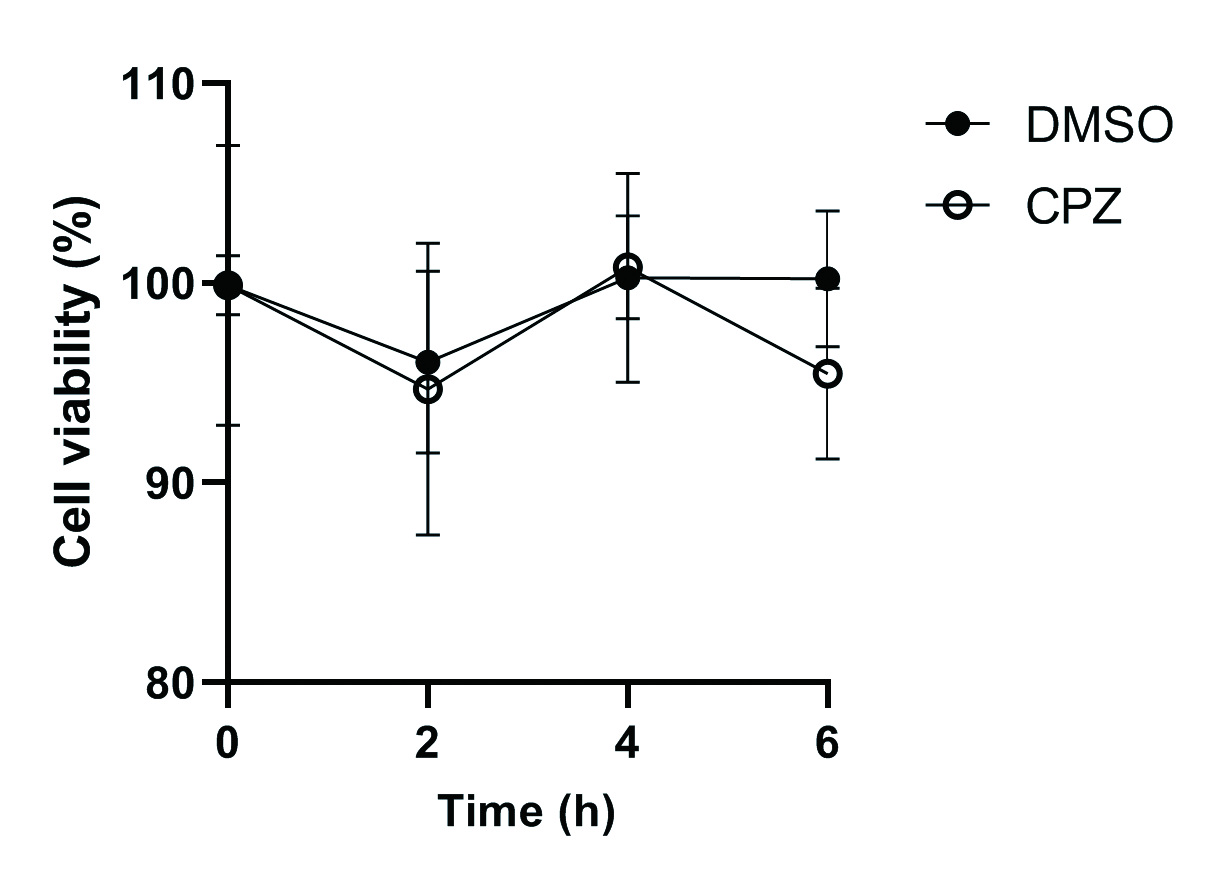

Supplement: Supplementary file 2 — Figure S1 [file 41419_2021_4450_MOESM2_ESM.tif]

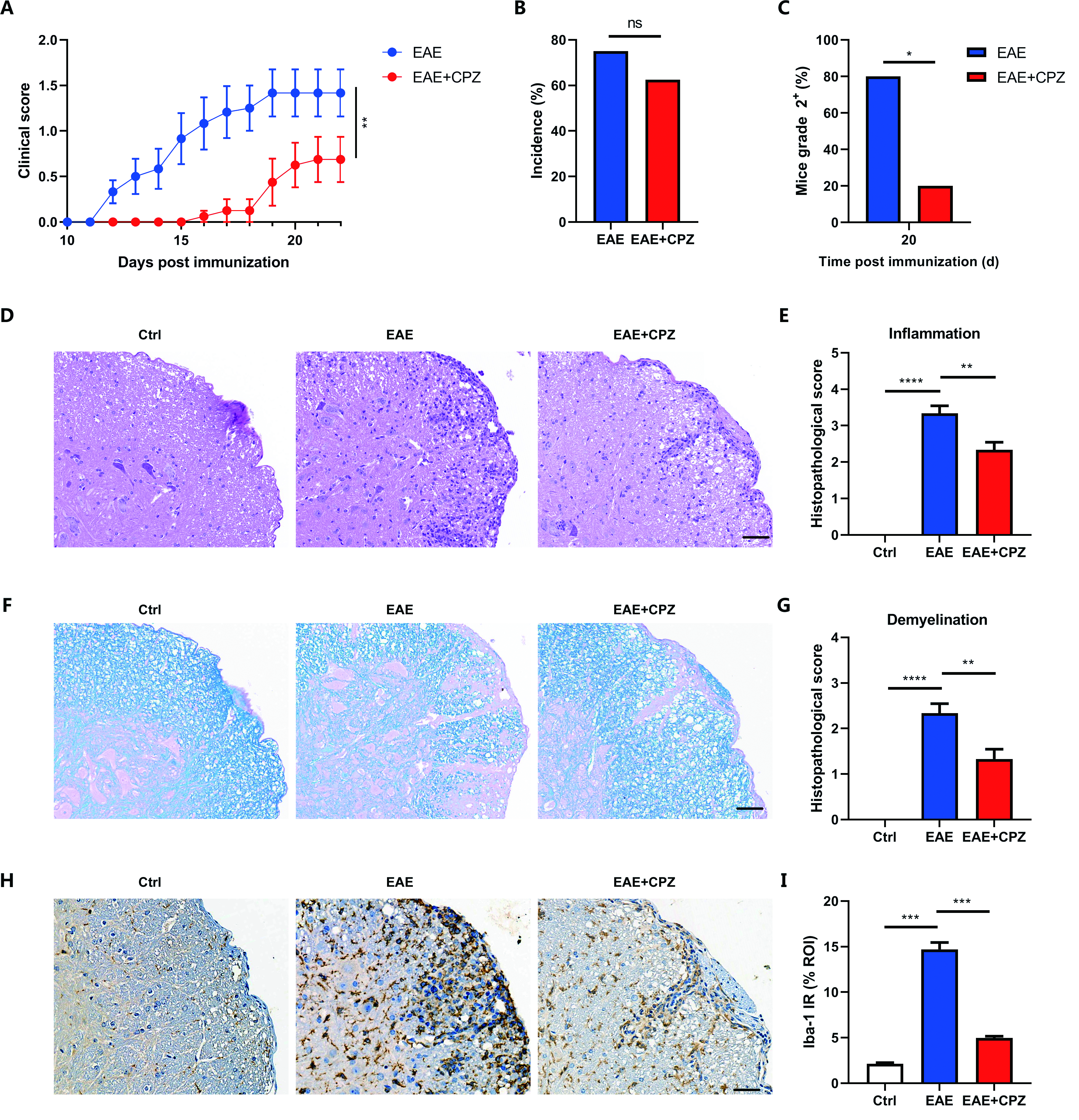

Supplement: Supplementary file 3 — Figure S2 [file 41419_2021_4450_MOESM3_ESM.tif]

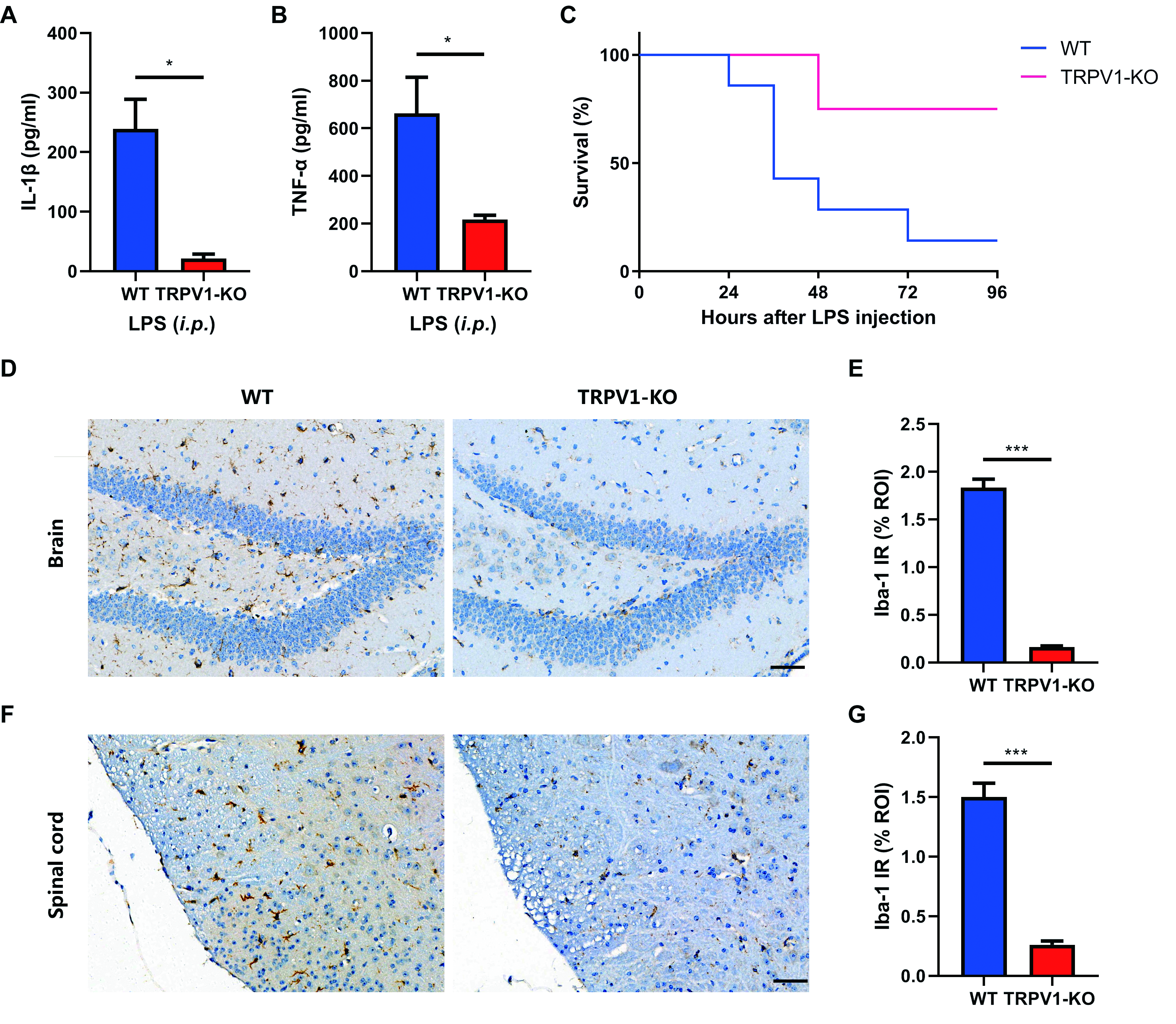

Supplement: Supplementary file 4 — Figure S3 [file 41419_2021_4450_MOESM4_ESM.tif]

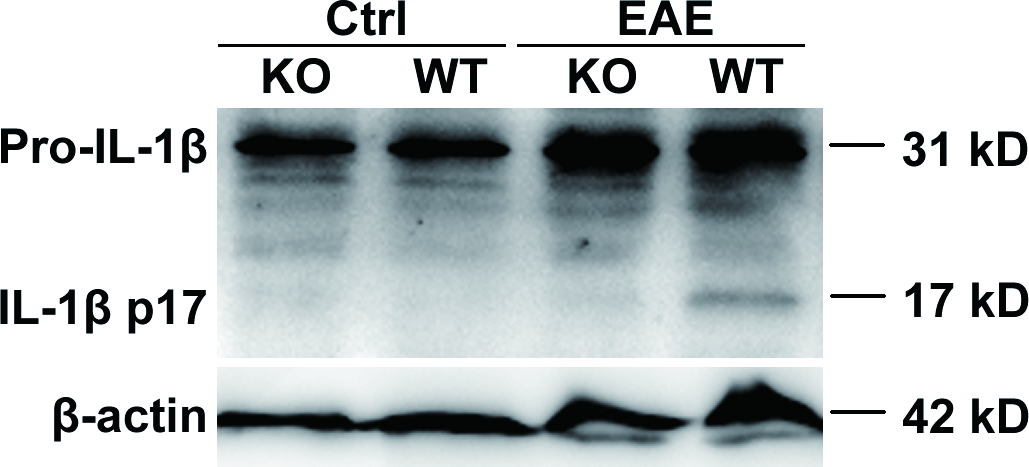

Supplement: Supplementary file 5 — Figure S4 [file 41419_2021_4450_MOESM5_ESM.tif]

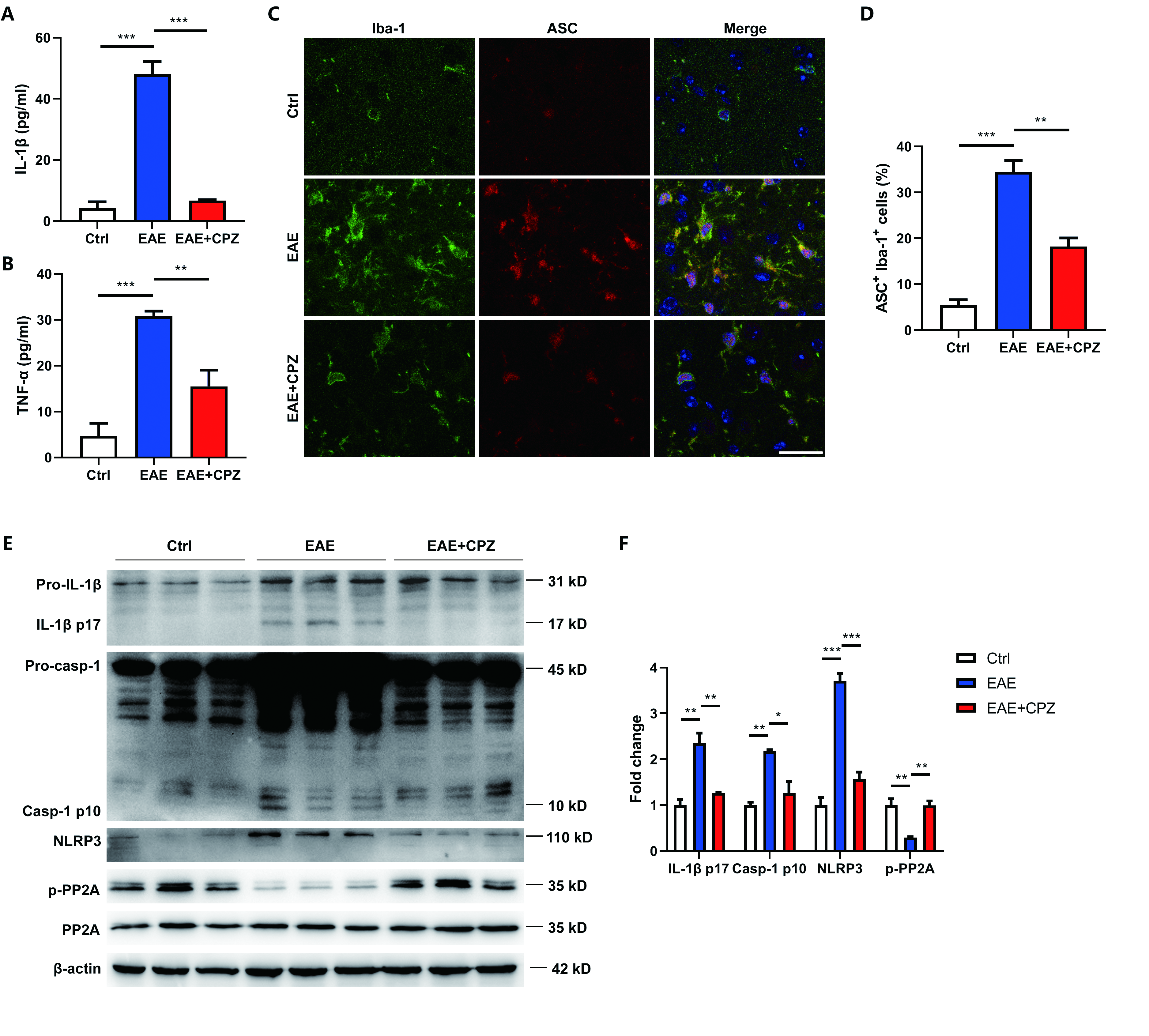

Supplement: Supplementary file 6 — Figure S5 [file 41419_2021_4450_MOESM6_ESM.tif]
